# A review of oral cannabinoids and medical marijuana for the treatment of chemotherapy-induced nausea and vomiting: a focus on pharmacokinetic variability and pharmacodynamics

**DOI:** 10.1007/s00280-017-3387-5

**Published:** 2017-08-05

**Authors:** Melissa E. Badowski

**Affiliations:** Chicago College of Pharmacy, University of Illinois, 833 S. Wood St M/C 886, Room 164, Chicago, IL 60612 USA

**Keywords:** Dronabinol, Cannabinoids, Chemotherapy-induced nausea and vomiting, Pharmacokinetics, THC

## Abstract

**Purpose:**

Oral cannabinoids (i.e., dronabinol, nabilone) containing the active component of marijuana, delta(Δ)9-tetrahydrocannabinol (THC), are available for the treatment of chemotherapy-induced nausea and vomiting (CINV) in patients with cancer who have failed to adequately respond to conventional antiemetic therapy. The aim of this article is to provide an overview of the efficacy, pharmacokinetics (PK), pharmacodynamics (PD), and safety of oral cannabinoids for patients with CINV.

**Methods:**

A PubMed search of the English-language literature available through 4 January 2017 was conducted to identify relevant articles for inclusion in the review.

**Results:**

Oral cannabinoids have been shown to have similar or improved efficacy compared with conventional antiemetics for the resolution of nausea and/or vomiting in patients with cancer. However, oral THC has high PK variability, with variability in oral dronabinol peak plasma concentrations (*C*_max_) estimated between 150 and 200%. A new oral dronabinol solution has decreased intraindividual variability (area under the curve) vs oral dronabinol capsules. Further, oral THC has a slower time to *C*_max_ compared with THC administered intravenously (IV) or by smoking, and a lower systemic availability than IV or smoked THC. The PD profile (e.g., “high”) of oral THC differs from that of IV or smoked THC in healthy individuals. Oral cannabinoids are associated with greater incidence of adverse effects compared with conventional antiemetic therapy or placebo (e.g., dizziness, hypotension, and dysphoria or depression).

**Conclusions:**

A new formulation of oral cannabinoids (i.e., dronabinol oral solution) minimized the PK/PD variability currently observed with capsule formulations.

## Introduction

An estimated 45 to 61% of patients with cancer experience chemotherapy-induced nausea and vomiting (CINV) [1, 2], which can occur as a result of chemotherapeutic agents and/or their metabolites activating neurotransmitter receptors in the gastrointestinal (GI) tract [e.g., 5-hydroxytryptamine type 3 (5-HT_3_)] and brain [e.g., neurokinin-1 (NK-1)] [3]. Acute CINV occurs within 24 h of initiating chemotherapy and mainly involves receptors in the GI tract, while delayed CINV occurs 1–5 days after starting chemotherapy and is primarily mediated by activation of receptors in the brain [3]. Delayed CINV occurs more frequently than acute CINV (58.4 vs 34.3%, respectively), and nausea is a more common component of the illness than vomiting (42 vs 20.8%) [1, 2, 4]. Oral and intravenous (IV) chemotherapeutic agents are classified in risk categories based on the frequency at which they may cause CINV in patients with cancer: high risk (>90%), moderate risk (30–90%), low risk (10–30%), and minimal risk (<10%) [5]. Patients receiving cancer chemotherapy categorized as moderate or high risk for nausea and vomiting typically remain at risk for ≥2 or ≥3 days, respectively, after receiving their final dose of chemotherapy [6]. Some agents are associated with a high emetogenic potential; emesis with cisplatin, for example, is generally most severe within 2–3 days of treatment, with symptoms present for 6–7 days, and potentially longer in some patients [5, 6].

The primary goal of antiemetic therapy is to prevent CINV [4, 7–9]. Patients with cancer receiving antiemetic therapy for the prevention of CINV according to guideline recommendations by the Multinational Association of Supportive Care in Cancer [10] were significantly more likely to experience complete response to antiemetic treatment (i.e., no nausea or vomiting, no use of rescue therapy) compared with patients who did not receive antiemetic therapy as recommended (59.9 vs 50.7%, respectively; odds ratio [OR] 1.4; 95% confidence interval [CI] 1.0–2.0; *P* = 0.03) [11]. A significantly greater percentage of patients with cancer receiving antiemetic therapy as recommended by guidelines had no CINV for 5 days after receiving a single dose of chemotherapy compared with patients receiving antiemetic treatment inconsistent with guidelines (53.4 vs 43.8%, respectively; *P* < 0.001) [12]. Thus, it is apparent that management of CINV according to published guidelines significantly improves this adverse effect of chemotherapy in patients.

In addition to the potential lack of adherence to antiemesis guidelines, additional barriers to optimal management of CINV include underestimation of the incidence of nausea and vomiting in patients receiving cancer treatment, as well as overestimation of the effectiveness of antiemetic treatment [1, 4, 13]. Fifty-four percent of patients with cancer receiving moderately emetic chemotherapy considered their antiemetic therapy to be effective (i.e., no nausea or vomiting, no rescue medication use) compared with a physician expectation of 75% [1]. Patient-related factors (e.g., age, race, income, education, alcohol use), cost of antiemetic therapy, and patient lack of adherence to antiemetic therapy may also negatively impact optimal management of CINV [14–16]. In some cases, patients fail to administer antiemetic therapy for prevention of CINV, instead choosing to wait for onset of nausea and vomiting [15].

Patients with poorly controlled CINV were more likely to experience a negative impact on daily function compared with patients with well-controlled CINV [2]. Additionally, results of an online survey of health care providers indicated that up to 32% of patients may experience a disruption of chemotherapy due to adverse events of nausea and vomiting [16]. Further, CINV has been associated with mean total direct costs (i.e., outpatient visits, emergency department visits, hospitalizations, medications) and indirect costs (i.e., work loss productivity, absenteeism) estimated at >$700 per patient during the 5 days following the first cycle of chemotherapy [2]. Thus, overcoming health care- and patient-related factors associated with poor control of CINV may impact outcomes in patients with cancer (e.g., improved quality of life, decreased costs).

The main psychoactive component of marijuana (*Cannabis sativa*) is delta(Δ)9-tetrahydrocannabinol (THC), which binds to cannabinoid receptors types 1 and 2 (CB_1_, CB_2_) that are located throughout the body, including in the brain (CB_1_) and the immune system (CB_2_) [17, 18]. Activation of CB_1_ by THC can have medically desirable effects, such as decreasing the incidence of nausea and vomiting [18]. Medical marijuana has been approved for use by approximately half of the states in the United States, although its use remains controversial [17]. However, synthetic pharmaceutical-grade THC (i.e., dronabinol capsules, nabilone capsules, and dronabinol oral solution) is approved in the United States for the treatment of nausea and vomiting associated with cancer chemotherapy in patients who failed to adequately respond to conventional antiemetic therapy [19–21]. Further, oral cannabinoids are recommended in guidelines for patients with cancer with breakthrough nausea and vomiting [8, 9]. Dronabinol is also indicated for the treatment of anorexia associated with weight loss in patients with AIDS. The aim of this review article is to provide an overview of the efficacy, pharmacokinetics (PK), pharmacodynamics (PD), and safety of oral cannabinoids (i.e., dronabinol, nabilone) for the treatment of patients with cancer and CINV; the limited data available for the PK, PD, efficacy, and safety of medical marijuana are also presented in the context of CINV.

## Materials and methods

A PubMed search of English-language articles available through 4 January 2017 was conducted to identify relevant articles for inclusion using the search terms “Δ9-tetrahydrocannabinol”, “cannabinoid”, “dronabinol”, “nabilone”, “marijuana”, “cancer”, “chemotherapy-induced nausea and vomiting”, “pharmacokinetics”, “pharmacodynamics”, “efficacy”, and “safety”. Reference lists from identified articles were used to identify additional publications for inclusion. Guidelines for antiemesis in patients with cancer were identified either through PubMed and/or the website of the medical society involved in development of these guidelines [e.g., National Comprehensive Cancer Network (NCCN; nccn.org)].

## Results

### Efficacy, pharmacokinetics, and safety of oral cannabinoids

#### Efficacy of oral cannabinoids

The first placebo-controlled study demonstrating the efficacy of THC for the treatment of CINV in patients with cancer was published in 1975 [22]. Since publication of this initial report, numerous clinical studies have examined the antiemetic efficacy of oral dronabinol or nabilone capsules for the treatment of patients with CINV, with two meta-analyses reporting significant improvements with cannabinoids compared with conventional antiemetic therapy [23, 24], and one meta-analysis finding that, while the antiemetic efficacy of cannabinoids was favored, compared with the antiemetic prochlorperazine for the resolution of nausea, vomiting, or nausea and vomiting (Table 1), the findings did not achieve significance [25]. However, studies included in these meta-analyses differed in methodology (e.g., crossover study, blinding), discontinuation rates, sample sizes, timing of drug administration, tumor type, and chemotherapeutic agent(s) used [23–25]. Further, meta-analyses differed not only in the specific outcomes analyzed (e.g., antiemetic efficacy vs resolution of nausea or vomiting, or both), but also in the specific studies included in the evaluation of a particular efficacy outcome (i.e., number of studies) and whether cannabinoids were evaluated individually or by drug class [23–25].

**Table 1:** Summary of antiemetic efficacy of pharmaceutical cannabinoids in patients with CINV

Treatments	Studies, *n* (no. of patients)	Statistical significance of treatments		
		Resolution of nausea and vomiting, RR (95% CI)	Resolution of nausea, RR (95% CI)	Resolution of vomiting, RR (95% CI)
Dronabinol or nabilone vs prochlorperazine [25]	9 (*n* = 1221)	2.0 (0.7–5.4)	1.5 (0.7–3.2)	1.1 (0.9–1.4)
Dronabinol vs prochlorperazine [23]	5 (*n* = 325)	0.7 (0.5–1.0); *P* = 0.03; NNT = 3.4	NR	NR
Nabilone vs prochlorperazine, alizapride, or domperidone [23]	6 (*n* = 277)	0.9 (0.7–1.1); *P* = 0.2	NR	NR
Dronabinol or nabilone vs prochlorperazine or alizapride [24]	13 (*n* = 422)	NR	1.4 (1.2–1.6); NNT = 6.4	1.3 (1.1–1.5); NNT = 8.0

Data presented for oral cannabinoids approved in the US for the treatment of CINV (i.e., dronabinol and nabilone)

*CI* confidence interval, *CINV* chemotherapy-induced nausea and vomiting, *NNT* number needed to treat, *NR* not reported, *RR* relative risk

In addition, a pooled analysis of 14 studies indicated that a significantly greater percentage of patients preferred cannabinoids compared with conventional antiemetics for the treatment of CINV [61 vs 26%, respectively; relative risk (RR) 2.4; 95% CI 2.1–2.8; number needed to treat, 2.8] [24]. These data were confirmed by a 2015 meta-analysis of nine studies that also reported patients preferred cannabinoids to conventional antiemetic agents (RR 2.8; 95% CI 1.9–4.0) [25]. Reasons for these similar findings were not provided, but the results may be antiemetic-dependent, as there were no differences between patient preference for cannabinoids over metoclopramide or chlorpromazine in single studies with a small number of participants (*N* = 40 and *N* = 64, respectively) [25].

Medical use of marijuana is controversial and no clinical trials have been conducted to date to compare the antiemetic efficacy of medical marijuana with the conventional antiemetic agents recommended as first-line therapy by the NCCN Clinical Practice Guidelines in Oncology for Antiemesis [17, 26]. Medical marijuana is currently not recommended by the NCCN for antiemesis in CINV [26]. However, given that approximately half of all states in the United States have approved the use of medical marijuana, health care providers are increasingly likely to encounter patients interested in receiving medical marijuana for antiemesis [17].

#### Variability in pharmacokinetics and pharmacodynamics of cannabinoids

Lack of antiemetic efficacy (i.e., failure to decrease incidence of CINV) of oral THC was initially reported in patients with sarcoma receiving chemotherapy. In these patients, the lack of antiemetic efficacy was thought to possibly be associated with the type of chemotherapeutic agent administered [27]. However, absorption of THC was also highly variable, with decreased incidence of nausea and vomiting associated with higher drug plasma concentrations of THC (50% incidence at >5 ng/mL vs 83% at <5 ng/mL). Subsequent studies of orally administered THC have confirmed high PK variability in healthy individuals (Table 2) [28–33]. Oral absorption of dronabinol is high (90–95%), but slow and variable [34]. Peak plasma concentrations of dronabinol and its metabolites have been observed at ~2 h postdose with dronabinol capsule in healthy individuals [31, 32]. Variability in peak plasma concentrations (*C*_max_) was estimated between 150 and 200% [28]. In healthy individuals currently reporting cannabis use, administration of supratherapeutic doses of THC (i.e., 75–90 mg) were associated with high interindividual variability [*C*_max_ range 9.0–127.1 ng/mL; time to *C*_max_ (*T*_max_) range 1–12 h] [33].

**Table 2:** Summary of pharmacokinetics of oral Δ9-THC in healthy individuals

Study design and population	Dose	*C*_max_ (ng/mL)	*T*_max_	AUC
Part 1: DB, DD, 2-way CO (≥2-wk washout) Part 2: R, DB, PBO-C, 3-way dose escalation (≥2-wk washout) [28] Part 1: *n* = 12; Part 2: *n* = 9; participants from Part 1 could continue to Part 2 of the study	Part 1: Δ9-THC 5 mg Part 2: Δ9-THC 6.5 mg, 8 mg, or PBO	Mean (% CV): 5 mg: 2.9 (51) 6.5 mg: 4.4 (42) 8 mg: 4.7 (62)	Mean (% CV): 5 mg: 56.0 min (73) 6.5 mg: 39.3 min (20) 8 mg: 43.6 min (26)	AUC_0–∞_ (min × ng/mL); mean (% CV): 5 mg: 188.7 (40) 6.5 mg: 286.6 (36) 8 mg: 377.2 (46)
DB, PBO-C, CO (1-wk washout) [29] (*N* = 24)	Single-dose Δ9-THC 2.5 mg	Mean (range): 3.2 (0.7–8.0)	Mean (range): 63.6 min (30–183)	NR
R, DB, DD, PBO-C, CO (2-wk washout) [30] Individuals ≥65 y (*N* = 12)	Single-dose Δ9-THC 3 mg, 5 mg, 6.5 mg, or PBO	Mean (range): 3 mg: 1.4 (0.5–3.5) 5 mg: 3.2 (1.5–7.0) 6.5 mg: 4.6 (2.1–8.6)	Mean (range): 3 mg: 0.9 h (0.7–0.9) 5 mg: 0.9 h (0.7–0.9) 6.5 mg: 0.7 h (0.7–0.9)	AUC_0–2 h_ (h × ng/mL); mean (range): 3 mg: 1.7 (0.8–4.1) 5 mg: 2.6 (1.0–7.6) 6.5 mg: 3.5 (1.3–11.4)
R, DB, PBO-C, CO [31] Cannabis-naïve individuals (*N* = 12)	Single-dose Δ9-THC 20 mg or PBO	Mean (SE): 7.2 (2.0)	2 h	NR
Study design not defined [32] (*N* = 6)	Δ9-THC 20 mg	Mean (SE): 7.9 (3.6)	2.3 h	NR
SB, PBO-C, CO, multiple dose escalation [33] Regular users of cannabis (*N* = 7)	Single-dose Δ9-THC increasing by 15 mg with each dose, up to maximum 90 mg, or PBO	30 mg (mean): 9.7 90 mg (range): 9.0–127.1	Overall median: 3.3 h 75 and 90 mg (range): 1–12 h	NR

Pharmacokinetic values presented as reported in each publication. Variability of the estimate not presented uniformly across studies

*AUC* area under the concentration–time curve; *AUC*_*0*–*2h*_ area under the concentration–time curve from time 0–2 h; *AUC*_*0*–∞_ area under the concentration–time curve from time 0 extrapolated to infinity; *Δ9*-*THC* delta(Δ)9-tetrahydrocannabinol; *C*_*max*_ maximum plasma concentration; *CO* crossover; *CV* coefficient of variation; *DB* double-blind; *DD* double-dummy; *NR* not reported; *PBO* placebo; *PBO*-*C* placebo-controlled; *R* randomized; *SB* single-blind; *SE* standard error; *T*_*max*_ time to maximum plasma concentration

A new tablet formulation of THC designed to improve drug uptake demonstrated a more rapid *T*_max_ in healthy individuals, with interindividual variability in *C*_max_ ranging from 42 to 62% [28]. In one study, peak plasma levels of oral THC (e.g., tablet, capsule) were lower and were achieved over a longer duration compared with IV THC, which reached peak plasma concentrations within 20 min of administration (*C*_max_, 62 ng/mL) [32]. In this study, participants reported a maximal “high” feeling, a subjective psychological effect of THC use, 30–50 min after IV administration, compared with 2.5–3 h after oral THC administration. By further comparison, participants who smoked marijuana reported a maximal “high” feeling at 30–90 min postdose, long after *C*_max_ was achieved. These physiologic effects may be mediated by the major metabolites of THC, including 11-hydroxy-Δ9-tetrahydrocannabinol (11-OH-THC) and 11-nor-9-carboxy-Δ9-tetrahydrocannabinol (THC-COOH) [32, 35], which were shown to have a longer *T*_max_ than THC when marijuana was smoked (i.e., *T*_max_ for a marijuana cigarette containing 3.55% THC: THC, 0.14 h; 11-OH-THC, 0.2 h; THC-COOH, 1.35 h) [36]. Systemic availability of oral THC is lower than that of smoked THC (4–12% vs 8–24%, respectively) [34, 37], as the systemic availability of oral THC is limited by extensive first-pass hepatic metabolism [35].

With regard to medical marijuana, dosing of smoked marijuana is variable, given interindividual differences in frequency and depth of inhalations, and the type of cannabis selected, as cannabinoid content varies by blend [28, 29]. High interindividual PK variability was demonstrated in a study of healthy individuals smoking low- and high-dose cannabis (i.e., containing 1.75 and 3.55% THC, respectively) [36]. The smoking protocol of this study included a 2-s inhalation, 10-s hold period, and 72-s exhalation and rest period for a total of 8 puffs over 11.2 min. Peak plasma concentrations of THC were observed 8.4 min after the first puff, with a mean *C*_max_ of 84.3 ng/mL (range 50–129 ng/mL) for low-dose cannabis and 162.2 ng/mL (range 76–267 ng/mL) for high-dose cannabis; THC plasma concentrations then decreased rapidly to 17.3 and 29.7 ng/mL, respectively, after 30 min.

Inhaling vaporized THC reduced exposure to harmful byproducts produced by smoking cannabis [26, 38]. Peak plasma concentrations of THC after inhalation of low- or high-dose vaporized cannabis (2.9% THC, 46.5 µg/L; 6.7% THC, 62.1 µg/L) were achieved within 10 min of administration in healthy regular users of cannabis [38]. Interindividual PK variability was observed with inhalation of vaporized THC, which was attributed, in part, to rate and depth of inhalation and time THC was held in the lungs of participants, as well as factors associated with delivery of vaporized THC, including heating temperature, number of balloon fills, and the amount and type of cannabis used [38].

**Fig. 1:**
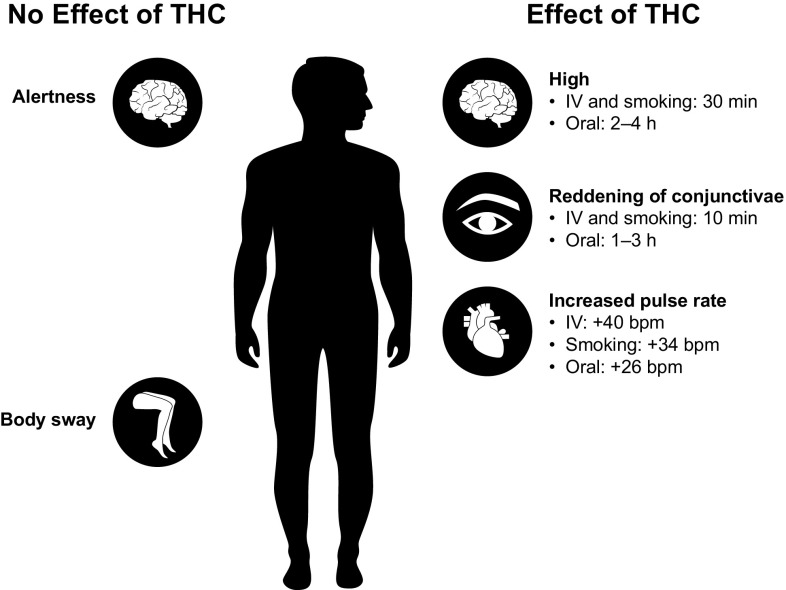
Variability in effects of Δ9-tetrahydrocannabinol on pharmacodynamics by route of administration in healthy individuals [30, 37, 39]. *bpm* beats per minute, *IV* intravenous, *THC* delta(Δ)9-tetrahydrocannabinol

The PD of oral THC is variable and differs from that of smoked or IV THC (Fig. 1) [30, 37, 39]. The PK/PD profile of orally administered THC (i.e., cookie) was shown to differ from that of smoked and IV administration of the drug in individuals with prior cannabis exposure [39]. Maximal feeling of “high” was achieved 30 min after smoking or IV administration of THC, and declined to baseline levels after 4 h; in contrast, after oral administration of THC, maximal feeling of “high” was slower in onset (i.e., 2–4 h), with a decline to baseline levels after 6 h. Peak plasma concentrations were achieved within 3 min following smoking or IV administration, but within approximately 1 h with oral administration. Plasma THC concentrations and the degree of “high” experienced by participants had high intra- and interindividual variability. Further, clinical signs of cannabis intoxication (e.g., reddening of conjunctivae, increased pulse rate) differed between smoked and IV administration vs oral administration [37]. Reddening of the conjunctivae reached a maximum effect by 10 min following smoking and IV administration, compared with a maximal effect observed 1–3 h after oral administration. In general, reddening of conjunctivae occurred with plasma THC concentrations >5 ng/mL, even in the absence of feeling “high.” The median increase from baseline in pulse rate was comparable between smoking and IV administration: an increase of 34 beats per minute (bpm) was observed with a median THC concentration of 45 ng/mL obtained via smoking, vs 40 bpm with a median plasma concentration of 100 ng/mL via IV administration. The effect of oral administration on pulse rate was lower compared with smoking and IV administration (26 bpm with a median THC concentration of 4.5 ng/mL). Pulse rate often returned to baseline or below while plasma concentrations remained >5 ng/mL and patients still reported feeling “high” [37]. Thus, it is apparent that plasma THC concentrations >5 ng/mL correlate better with reddening of conjunctivae than pulse rate, although both are considered clinical indicators of cannabis intoxication.

Single-dose oral administration of THC tablets (3.0, 5.0, or 6.5 mg) in healthy adults ≥65 years of age indicated no association between plasma THC concentrations and eyes open-body sway scores [*P* = 0.1; determined by SwayStar™ (BESTec-etp Freiburg GmbH, Freiburg, Germany), a device used to measure body movement when standing with eyes open or closed]. However, the eyes open-body sway scores were associated with plasma concentrations of the THC metabolites 11-OH-THC and THC-COOH [30]. This is a potentially clinically relevant finding in the context of falls, which are a primary cause of morbidity and mortality in the elderly [40]. In the same study of THC tablets, alertness scores were not associated with plasma concentrations of THC (*P* = 0.5), or its metabolites 11-OH-THC (*P* = 0.7) and THC-COOH (*P* = 0.8) [30].

**Fig. 2:**
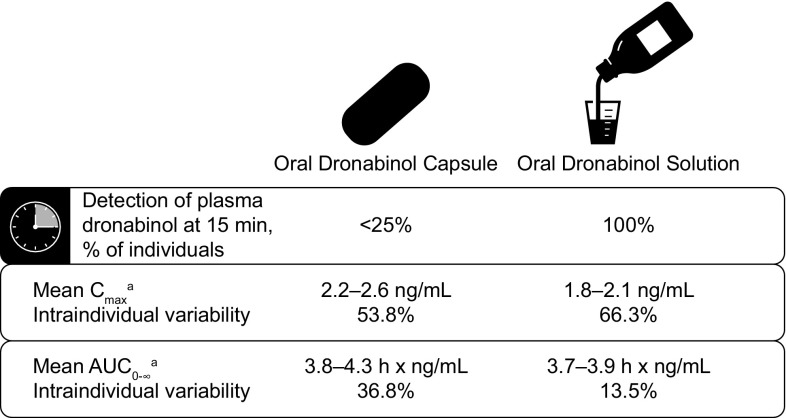
Variability in pharmacokinetics of oral dronabinol capsule 5 mg and oral dronabinol solution 4.25 mg following single-dose administration in healthy individuals [41]. ^a^Data for 2 replicates. *AUC*_*0*–∞_ area under the concentration–time curve from time 0 extrapolated to infinity, *C*_*max*_ maximum plasma concentration, *SD* standard deviation

The high PK variability of oral THC tablets and capsules may compromise accurate and consistent dosing of dronabinol [28]. The oral dronabinol solution formulation, approved by the US Food and Drug Administration (FDA) in July 2016, has been shown to have less variability, with drug detected in plasma in 15 min in 100% of individuals receiving this formulation compared with <25% of individuals receiving dronabinol capsule (Fig. 2) [41]. Further, the intraindividual variability in the mean area under the concentration–time curve from time 0 extrapolated to infinity (AUC_0–∞_) was decreased with oral dronabinol solution compared with the capsule. These findings have important clinical implications for patients with CINV, as patients may derive therapeutic benefit faster with oral dronabinol solution than with dronabinol capsule. Further, decreased intraindividual variability with oral dronabinol solution vs capsule may minimize the need to individualize dosing to obtain optimal therapeutic effects [19]. However, if needed, individualized dosing based on body surface area and titration of dosing to achieve clinical benefit is supported by current US labeling [21].

#### Safety of cannabinoids

Cannabinoids are associated with a number of potential adverse effects, including a “high” feeling, euphoria, disorientation, and depression [26]. Adverse effects of cannabinoids on non–central nervous system functions (e.g., tachycardia, reddening of conjunctivae, decreased GI motility) are attributed to the ubiquitous localization of cannabinoid receptors throughout the body [42]. Results of a meta-analysis showed that patients receiving oral dronabinol or nabilone capsules had greater incidence of adverse effects compared with those receiving conventional antiemetic therapy or placebo: dizziness, 49 vs 17%, respectively; hypotension, 25 vs 11%; dysphoria or depression, 13 vs 0.3%; hallucinations, 6 vs 0%; and paranoia, 5 vs 0% [24]. Medical use of cannabinoids (including oral dronabinol and nabilone capsules) for various conditions, including CINV, chronic pain, spasticity related to multiple sclerosis or paraplegia, human immunodeficiency virus/AIDS, and sleep disorder, was associated with a greater risk of adverse effects compared with an active comparator or placebo in 62 studies [43]. Overall, the most common adverse effects following administration of cannabinoids included disorientation (OR 5.4; 95% CI 2.6–11.2), dizziness (OR 5.1; 95% CI 4.1–6.3), euphoria (OR 4.1; 95% CI 2.2–7.6), confusion (OR 4.0; 95% CI 2.1–8.0), and drowsiness (OR 3.7; 95% CI 2.2–6.0). Although data are limited, single-dose dronabinol oral solution was shown to be generally well tolerated, with nausea, dizziness, somnolence, and headache the most common adverse events reported by healthy volunteers [41].

### Considerations for oral cannabinoids compared with medical marijuana

Numerous routes of administration are available for patients with cancer receiving medical marijuana, including smoking, oral (e.g., cookie, candy, beverages), and mucosal [44–46]. In contrast with oral cannabinoids (i.e., dronabinol, nabilone), medical marijuana is not currently regulated by the FDA [47]. Thus, there is currently a lack of standardization regarding dosing and potency across available medical marijuana formulations; additionally, the potential for food safety issues cannot be excluded for users of oral products (i.e., foodstuffs, beverages) [44–46, 48]. While edible medical marijuana products are required to have child-resistant packaging and labeling, unintentional pediatric exposures may still occur [47, 49]. Use of smoked marijuana for medical purposes by patients with cancer has several limitations, including a patient’s inability to tolerate smoked marijuana due to taste or the potential for airway obstruction, which may result from inflammation of the airway following smoking [50, 51]. Medical marijuana may also increase the risk for atrial fibrillation, myocardial infarct, and chronic bronchitis [26]. Further, patients who are immunocompromised may risk additional immunosuppression (e.g., by suppressing lymphocyte proliferation) following use of medical marijuana [26, 52]. In addition, insurance will generally cover the costs associated with use of cannabinoids approved by the FDA, but not medical marijuana. Overall, additional studies comparing the safety and efficacy of oral cannabinoids with various formulations of medical marijuana are needed.

## Discussion

Management of patients receiving cancer chemotherapy includes preventing the treatment-related adverse effects of nausea and vomiting [9]. Most clinical studies of antiemetic agents in patients affected by CINV have focused on prevention, rather than treatment, of nausea and vomiting, supporting earlier intervention rather than delayed use [53]. However, given that many patients are at risk for CINV for days after receiving the last dose of chemotherapy [6], identification of effective therapies for the treatment of current CINV symptoms is important. A number of factors play a role when considering therapeutic options for controlling CINV, including tolerability of antiemetic therapy, patient setting (i.e., inpatient, outpatient), and patient preference [e.g., route of administration (oral, IV)] [9]. Oral cannabinoids were initially approved for the treatment of CINV in the 1980s; however, current use of this drug class for the treatment of CINV is limited by occurrence of adverse effects, including dizziness, dry mouth, and drowsiness, in some patients [43, 53]. Further, oral cannabinoids were, until recently, only available as capsules or tablets. The recent approval of medical marijuana in a number of states warrants additional well-designed studies examining efficacy and safety in patients with CINV.

The recently approved oral dronabinol solution not only has a favorable PK profile, but plasma levels of dronabinol were detected within 15 min in all individuals examined [21, 41]. Patients with CINV may benefit from these favorable attributes of oral dronabinol solution, with the potential for faster onset of relief of nausea and vomiting. Further, oral dronabinol solution is an easy-to-swallow formulation for patients with CINV that is administered using a 1-mL oral syringe provided with the medication; of note, labeling indicates that oral dronabinol solution should be administered with 6–8 oz of water [21]. Finally, while use of oral cannabinoids is limited by the potential for adverse effects, patients with CINV refractory to treatment with other antiemetic agents may benefit from administration of dronabinol or nabilone for treatment of CINV [25].

Some practical considerations should be noted for oral dronabinol solution, including that the agent is available as an unflavored 5 mg/mL formulation with a clear, pale yellow to brown color [21]. Dosing should be calculated based on patient body surface area (m^2^) multiplied by 4.2 mg/m^2^ and rounded to 0.1 mg [21]. Patients should receive an initial dose of dronabinol oral solution on an empty stomach (i.e., 30 min before eating) 1–3 h prior to receiving chemotherapy; subsequent doses can be administered, without considering timing of food consumption, every 2–4 h after chemotherapy (up to 4–6 doses daily) [21]. Dronabinol oral solution is contraindicated in patients receiving disulfiram- or metronidazole-containing agents currently, or within the past 14 days, as patients may experience a disulfiram-like reaction (e.g., abdominal cramping, nausea, vomiting) [21]. Patients with a history of seizures should be monitored, given that seizures and seizure-like activity have been reported with dronabinol [21]. Patients may experience new or worsening nausea, vomiting, and abdominal pain, the occurrence of which requires a decrease in dosing or discontinuation of dronabinol oral solution [21]. In addition, dronabinol oral solution should not be administered to patients with a history of psychiatric events [21].

## Conclusions

Oral cannabinoids (i.e., tablets, capsules) are efficacious for the management of nausea and vomiting associated with chemotherapy treatment, but capsules are associated with an increased incidence of adverse effects compared with conventional antiemetic therapy [24, 26, 43]. However, patients have shown a preference for oral cannabinoid therapy (i.e., tablets, capsules) compared with some conventional antiemetics [24, 25]. The PK/PD profile of oral cannabinoids administered as capsules is highly variable and differs from that of THC smoked or administered by IV [30, 32, 37, 39]. Medical marijuana (i.e., smoked, vaporized, ingested) also has a variable PK profile, for reasons thought to be associated with individual differences in inhalation and/or type of cannabis being used [28, 29, 39]. The 2016 approval of dronabinol oral solution may reduce the PK variability currently observed with oral dronabinol capsule formulations and may provide patients with CINV the option of greater treatment flexibility. However, more studies examining the PK profile of THC-containing products in patients with CINV are warranted, as data for this specific population are currently lacking in the literature.
